# Stimulation of Replication Template-Switching by DNA-Protein Crosslinks

**DOI:** 10.3390/genes10010014

**Published:** 2018-12-27

**Authors:** Laura T. Laranjo, Julie A. Klaric, Leah R. Pearlman, Susan T. Lovett

**Affiliations:** Department of Biology and Rosenstiel Basic Medical Science Research Center, Brandeis University, Waltham, MA 02454-9110, USA; llaranjo@brandeis.edu (L.T.L.); jklaric93@brandeis.edu (J.A.K.); leahreneepearl@brandeis.edu (L.R.P.)

**Keywords:** DNA replication, DNA repair, genetic recombination, mutagenesis

## Abstract

Covalent DNA protein crosslinks (DPCs) are common lesions that block replication. We examine here the consequence of DPCs on mutagenesis involving replicational template-switch reactions in *Escherichia coli.* 5-Azacytidine (5-azaC) is a potent mutagen for template-switching. This effect is dependent on DNA cytosine methylase (Dcm), implicating the Dcm-DNA covalent complex trapped by 5-azaC as the initiator for mutagenesis. The leading strand of replication is more mutable than the lagging strand, which can be explained by blocks to the replicative helicase and/or fork regression. We find that template-switch mutagenesis induced by 5-azaC does not require double strand break repair via RecABCD; the ability to induce the SOS response is anti-mutagenic. Mutants in *recB*, but not *recA*, exhibit high constitutive rates of template-switching, and we suggest that RecBCD-mediated DNA degradation prevents template-switching associated with fork regression. A mutation in the DnaB fork helicase also promotes high levels of template-switching. We also find that other DPC-inducers, formaldehyde (a non-specific crosslinker) and ciprofloxacin (a topoisomerase II poison) are also strong mutagens for template-switching with similar genetic properties. Induction of mutations and genetic rearrangements that occur by template-switching may constitute a previously unrecognized component of the genotoxicity and genetic instability promoted by DPCs.

## 1. Introduction

The DNA replication fork frequently encounters barriers such as DNA secondary structures, adducts (including crosslinks) and tightly-bound protein complexes. Bypass or removal of these barriers is necessary to sustain cell viability. Misrepair at these lesions can cause genetic changes, which in humans may lead to cancer, neurodegeneration and premature aging [1,2].

An emerging area of study concerns the tolerance and repair mechanisms that deal with collisions that occur between the replication machinery and DNA-protein crosslinks (DPCs) (reviewed in [3,4,5,6,7,8]). DPCs are produced either by exogenous sources of DNA damage, such as ionizing radiation and by several chemotherapeutic agents, or by endogenous DNA damage caused by reactive oxygen or nitrogen species or reactions with natural metabolic products, such as aldehydes. A number of normal cellular enzymes, such as topoisomerases and cytosine methyl-transferases, produce covalent complexes as intermediates in their reaction mechanisms. Trapping of these intermediates by certain enzyme inhibitors can promote covalent DPCs. It has been estimated that each mammalian cell carries a basal burden of 6000 DPCs [9] and the inability to repair these lesions leads to accelerated aging and cancer proneness [5,10,11,12,13,14].

Most studies of DPCs have examined cellular processes that contribute to tolerance or repair of DPCs; however, the consequence of DPCs on genetic stability has not been fully explored. We suspected that certain classes of mutation would be elevated because DPCs lead to replication stalling [3,4,15]. Copy-number variation and certain templated-genetic mutations are triggered by misalignments of the nascent strand with a template strand during DNA replication (reviewed in [16,17]). The likelihood of these events is enhanced by difficulties in DNA replication. We have found that inhibition of replication induces rearrangements that lead to local deletions and expansions of tandemly repeated sequences in the bacterium *Escherichia coli* [18,19]. In addition, certain base substitutions and frameshift mutations that occur by a replication template-switch mechanism at quasi-palindromic (QP) sequences are stimulated strongly by mild, sublethal replication inhibition [20,21].

Here we use *lacZ* genetic reporter strains of *E. coli* to measure the rate of template-switching associated with DPCs produced by cytosine methyl-transferase (induced with 5-aza-cytidine), topoisomerase II (induced by ciprofloxacin) or by reaction with aldehydes (induced with formaldehyde). We find that 5-azacytidine (5-azaC) at sublethal doses induces template-switching associated with replication, especially on the leading strand. 5-azaC-induced mutations on both strands require a function DNA cytosine methylase (negated in a *dcm* mutant); therefore 5-azaC likely stimulates template-switching via trapping of the Dcm protein:DNA covalent complex. Although homologous recombination via the RecA, RecBCD-double strand break (DSB) repair pathway aids tolerance of 5-azaC and cell survival at high doses, DSB repair is not required for mutagenesis associated with 5-azaC. Dependence of 5-azaC mutagenesis on intact *dnaE*, encoding the polymerase subunit of the DNA Pol III replicative complex, suggests that mutagenesis occurs during blocked chromosomal replication. Since mutation rates are constitutively high in *dnaB* and *recB* mutants, we argue that dissociation of the fork helicase and fork regression contribute to template-switching during replication. The SOS DNA damage response aids avoidance of template-switch mutations both constitutively and after treatment with 5-azaC. Other DPC-inducing compounds such as formaldehyde and ciprofloxacin act similarly to 5-azaC: they strongly induce template-switching at sublethal concentrations independent of RecABCD, more robustly on the leading-strand. Template-switching, with the potential to promote genetic instability, is therefore likely to be strongly associated with DPCs.

## 2. Materials and Methods

### 2.1. Bacterial Strains, Growth Conditions, and Media

The bacterial strains used (Table 1) are isogenic derivatives of *Escherichia coli* K-12 MG1655 [22], constructed by P1 *vir*A transduction [23]. Standard growth medium was Luria broth (LB, Lennox formulation) medium, consisting of 1% Bacto-tryptone, 0.5% yeast extract, 0.5% sodium chloride and, for plates, 1.5% Bacto-agar. LB medium supplemented with 1% glucose, 2 mM calcium chloride and 1% agar for plates, was used to make P1 lysates and for transductions.

Antibiotics used in genetic selections included tetracycline (15 µg/mL) and kanamycin (60 µg/mL) (both from USB Products, Cleveland, OH, USA). Lac^+^ reversion mutants were selected on lactose minimal medium (56/2 salts [24], with 0.2% lactose (Sigma Aldrich, St. Louis, MO, USA), 0.001% thiamine (Sigma Aldrich), 2% agar (ThermoFisher, Sparks, MD, USA). X-gal (40 µg/mL) and IPTG (0.1 mM) (Both from Gold Bio, St. Louis, MO, USA) were included in lactose section medium to aid counting of colonies.

### 2.2. Disk Diffusion Assays

Lactose X-gal papillation plates, as in [21] but with a lower concentration of lactose at 0.5%, were used to detect reversion mutants in the disk diffusion assay. Cultures inoculated from single colonies in 1.5 mL LB medium were grown overnight at 37 °C with aeration. 100 μL of the culture (∼2 × 10^8^ cells) was spread on the plate. 5-azaC (Sigma Aldrich), ciprofloxacin (Cipro, Sigma Aldrich) or formaldehyde (ThermoFisher) were dissolved in water at the indicated concentrations. 5 μL was spotted onto 6.35-mm Whatman No. 1 filter disks (Whatman, Maidstone, UK) overlaid on the bacterial lawn. Controls included 5 μL of sterile water spotted on a second disk. Plates were incubated at 37 °C for 1–2 days. 

### 2.3. Survival Assays

Cultures inoculated from single colonies in 1.5 mL LB medium were grown overnight at 37° with aeration. The culture was diluted 1:100 in fresh 1.5 mL LB medium and grown for two hours at 37 °C with aeration. After this incubation, the entire 1.5 mL culture was spun down at 9000 rpm for two minutes and the cell pellet was resuspended in 150 μL of fresh LB medium. The sample was split evenly among tubes with 1.5 mL of LB medium with the concentrations of drug indicated in each experiment. The tubes were grown for another two hours at 37 °C with aeration. The entire culture was recovered by microcentrifugation, washed twice with 1 mL of 1× 56/2 buffer, and resuspended in 200 μL of 56/2 buffer. 20 μL of the cells was subjected to serial dilution and plated on LB plates to determine the CFU (colony forming units)/mL.

### 2.4. Mutation Assays

We used previously constructed and validated *lacZ* mutational reporters, QP5 and QP6 [20] that revert to Lac^+^ by a 4-bp deletion, templated by the one arm of a 18 bp quasipalindrome in the *lacZ* gene. Cultures were inoculated from single colonies in 1.5 mL LB medium and grown overnight (<16 h) at 37 °C with aeration. The culture was diluted 1:100 in fresh 1.5 mL LB medium and grown for two hours at 37 °C with aeration, with and without DPC agents, 12.5 µg/mL 5-azaC, 25 ng/mL ciprofloxacin, or 1 mM formaldehyde. Entire culture was washed twice with 1 mL and resuspended in 150 μL of 56/2 buffer. A small fraction <1% of the cells was subjected to serial dilution and plating on LB plates to determine number of CFU/mL. The remainder were plated on minimal lactose medium and incubated for two days at 30 °C for temperate-sensitive strains and 37 °C for the remaining strains. Rate of reversion was assessed by a fluctuation analysis as previously described [20,25]. Rates were calculated with 95% confidence interval using the Ma-Sandri-Sarkar Maximum Likelihood method [26,27]. Rates were determined from at least six split cultures. QP revertants for at least 10 independent isolates were confirmed by PCR and DNA sequence analysis as described [20].

## 3. Results

### 3.1. 5-Azacytidine Induces DNA Cytosine Methylase-Dependent Mutagenesis at Quasipalindromes

**Figure 1:**
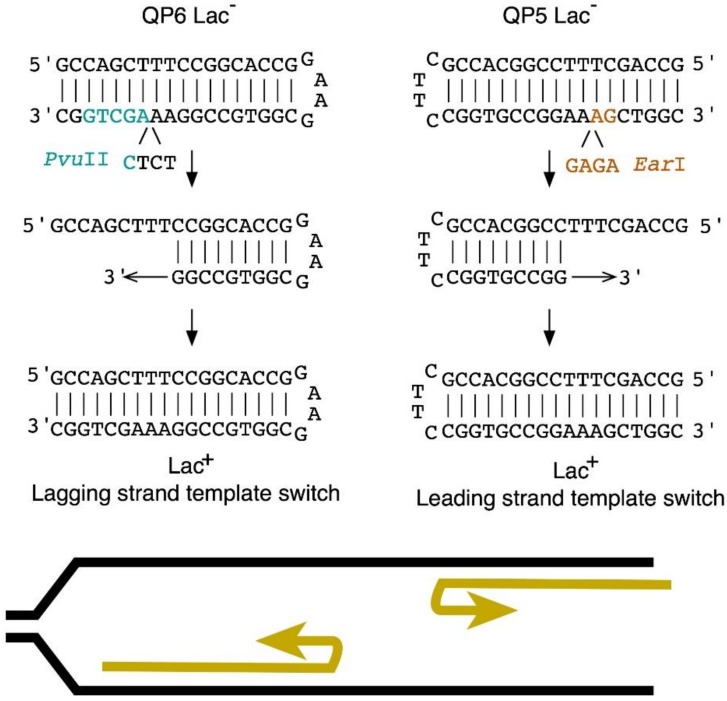
Template-switch reporter strains, QP5 and QP6. The sequence of the *lacZ* quasipalindrome is illustrated before and after Lac^+^ reversion. The template switch misalignment is diagrammed below for QP6 on the lagging strand of replication, at the left, and for QP5 on the leading strand of replication, at the right.

Template-switch mutations in an 18-bp quasipalindrome sequence were detected with previously developed mutation reporter strains, QP5 and QP6 (Figure 1). In these assays, deletion of a 4 bp insertion in the chromosomal *lacZ* locus reverts the strain to Lac^+^, concomitant with a loss of a diagnostic restriction site, *Ea*rI (QP5) or *Pvu*II (QP6) in *lacZ*. The 4-bp insertion site is on opposite sides of the inverted repeat, with QP5 reporting a replication template switch on the leading strand and QP6 that on the lagging strand (Figure 1).

**Figure 2:**
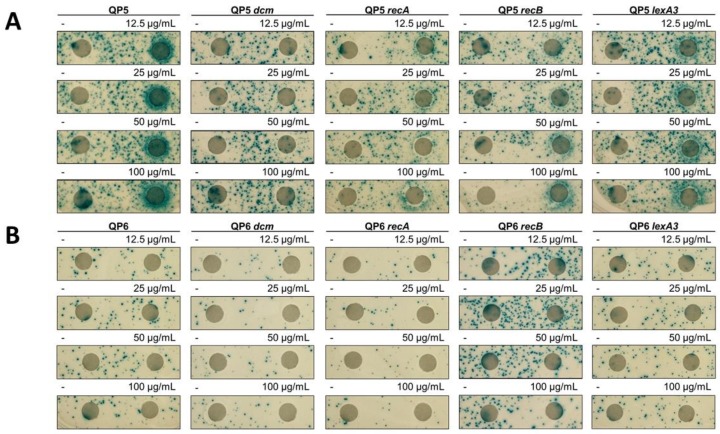
Mutator effect of 5-azacytidine. Disk diffusion assay for Lac reversion using QP5 (**A**) and QP6 (**B**) reporters ± *dcm*, *recA*, *recB*, *lexA3* and lactose X-gal papillation medium. Each disk on the right was saturated with 5 μL of 5-azacytidine at the indicated concentrations. The control disk on the left was spotted with 5 μL of water.

To test the mutagenic potential of different compounds over a wide range of concentrations, we employed a disk-diffusion papillation assay [20], in which Lac^+^ revertants from a lawn of mutation reporter strains are visualized on an X-gal plate. Paper disks were overlaid on the plate, with drugs (or water) subsequently spotted on the disk. The ring of blue Lac^+^ colonies of QP5 encircling the disk with 5-azaC indicates a mutagenic effect by 5-azaC (Figure 2). Deletion of *dcm*, (the gene encoding DNA cytosine methylase) in QP5 abolished the mutagenic effect of 5-azaC (Figure 2). Because 5-azaC traps the covalent complex formed between Dcm methyl transferase [29,30], dependence of mutability on Dcm suggests that the mutagenic effect derives from DPCs. We did not detect 5-azaC-induced mutagenesis with the QP6 reporter in the disk diffusion assay, at any of the doses tested (Figure 2), although more quantitative assays (see below) suggest an effect. This indicates that for template-switch mutations, 5-azaC may have a stronger mutagenic effect on the leading strand than the lagging strand.

**Figure 3:**
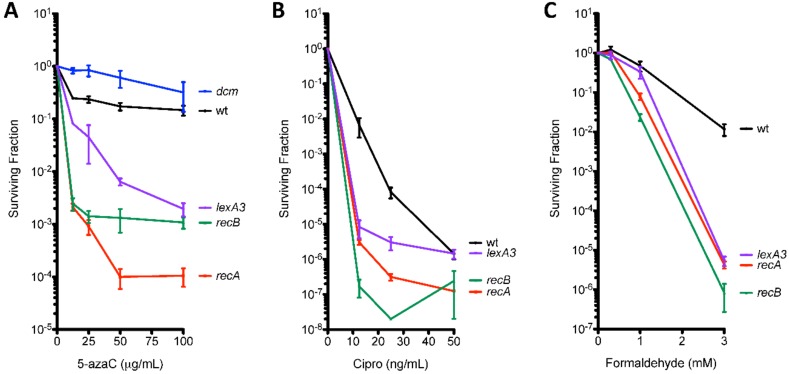
Survival curves. Survival of MG1655 (wt), *dcm*, *recA*, *recB*, and *lexA3* strains exposed to 5-azaC (**A**), ciprofloxicin (**B**) or formaldehyde (**C**). MG1655 strains were grown to exponential phase and then treated with drug for 2 h. Each point represents the mean survival of three to five biological replicates with error bars indicating the standard error of the mean. (wt: black, *dcm*: blue, *recA*: red, *recB*: green, *lexA3*: purple). Orange dotted lines indicate the doses used in the mutation rate assays.

**Figure 4:**
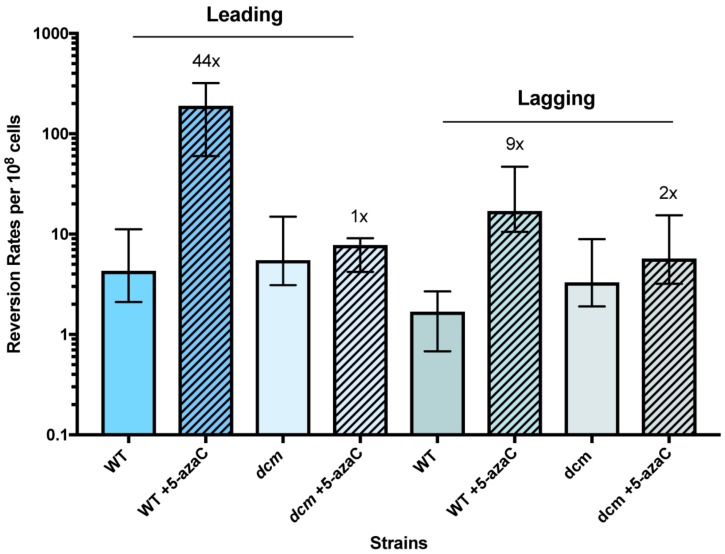
Reversion rates of *lacZ* QP alleles using leading and lagging strand reporters. Data shows reversion rates per 10^8^ cells from at least 6 multiple trials performed in different days of wild-type and *dcm* null strains. Rates are: 4.3, 5.5, 1.7, and 3.3 respectively. Rates of leading and lagging strand reporters with treatment with 12.5 µg/mL of 5-azacytidine are 190, 7.8, 20, and 5.7. Fold change comparing rates of reversion with and without exposure of 5-azacytidine is indicated above bars, and are 44, 1, 12 and 2, respectively.

The rates of template-switch mutations in an 18-bp quasipalindrome sequence were quantitated by Lac^+^ reversion assays using mutation reporter strains, QP5 and QP6. To test the effects of 5-azaC on template-switch mutagenesis we split independent cultures of QP5 and QP6 and added 12.5 µg/mL 5-azaC to one of these. After two h of incubation during which the cells were actively growing, the cells were harvested and the number of Lac^+^ revertants relative to total CFU was determined by serial dilution and plating. Although this 5-azaC treatment results in negligible loss of viability in otherwise wild-type (wt) strains (Figure 3). we observed a strong stimulation of mutation rate by 5-azaC: A 44-fold stimulation with the leading strand reporter QP5 and a 12-fold stimulation with lagging strand reporter QP6 (Figure 4). In QP5 and QP6 reporter strains in which the Dcm cytosine methylase gene has been deleted, mutation rates were not significantly elevated by 5-azaC treatment. Restriction digests and DNA sequence analysis from *lacZ* PCR products from independent revertants confirmed that that the 4 bp insertion had been lost, as predicted by a template switch mechanism. We conclude that 5-azaC treatment does indeed induce mutations on both strands but has a stronger effect on the leading strand than on the lagging strand.

### 3.2. 5-Azacytidine-Induced Mutagenesis Is Largely RecA-Independent and Does Not Require DSB Repair

**Figure 5:**
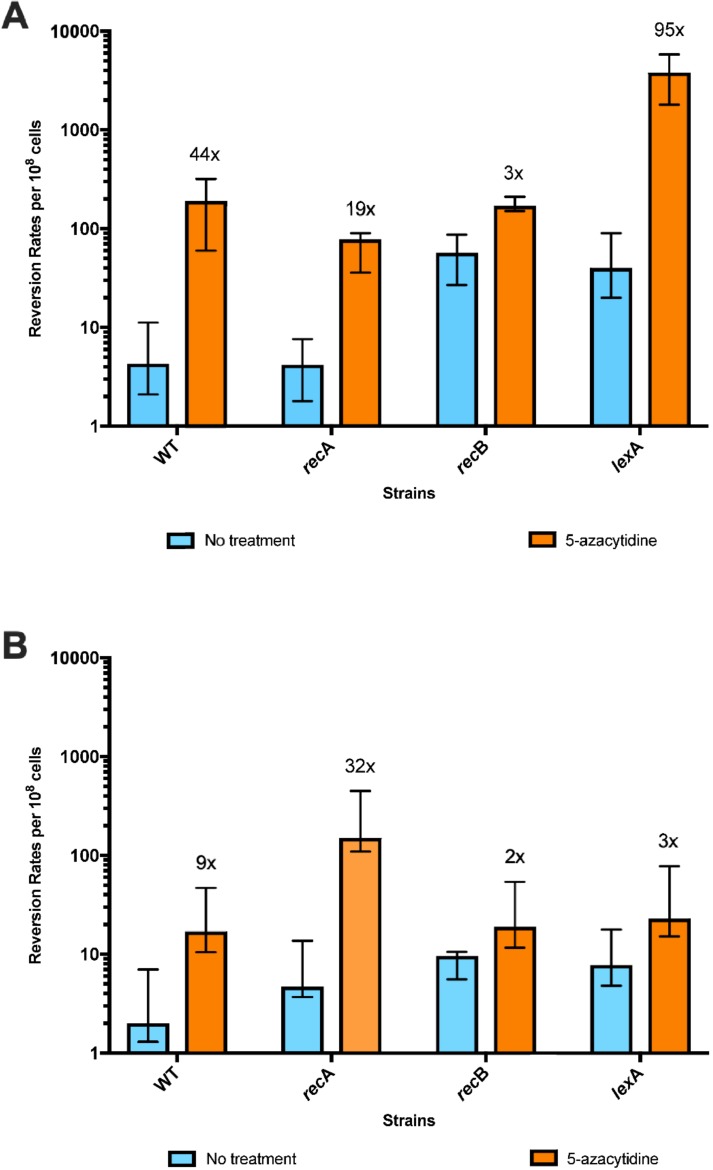
Reversion rates of *lacZ* QP5 and QP6 *recA*, *recB*, and *lexA3* mutational reporters after treatment with 5-azacytidine. Data show reversion rates per 10^8^ cells from at least 6 multiple trials performed on different days. Error bars represent 95% confidence interval. Reversion rates of *lac*Z QP5 *recA*, *recB*, and *lexA3* mutant strains with and without exposure of 12.5 ug/mL of 5-azacytidine. (**A**) Rates for QP5 reporters are 4.3, 190, 4.2, 78, 57, 170, 40, and 3800. Fold change comparing rates of reversion before and after exposure are 44, 19, 3, and 95, respectively. (**B**) Rates for QP6 reporters are 2, 17, 4.7, 150, 9.6, 19, 7.8, and 23. Fold change comparing rates of reversion before and after exposure are 9, 32, 2 and 3 respectively.

The ability to repair double-strand DNA breaks through the RecA RecBCD-dependent homologous recombination pathway has been shown to promote survival to 5-azaC [31,32,33,34,35]. We confirmed the *recA* and *recB* were required to sustain viability during a 2-h treatment with 5-azaC at various concentrations (Figure 3A). To test whether template-switch mutagenesis is occurring during DSB repair, we treated *recA* and *recB* derivatives of QP5 with 5-azaC and measured mutation rates. We observed a 19-fold stimulation of mutagenesis by 5-azaC in *recA* mutants (compared to 44-fold for *recA*^+^), showing that DSB repair is not absolutely required for template-switch mutagenesis induced by 5-azaC (Figure 5). RecA may be required some component of 5-azaC-induced template-switch mutagenesis, given the 2.5-fold lower yield; however, this small reduction may derive from the poor viability of *recA* mutants treated with 5-azaC (Figure 3). A similar effect is evident in the disk-diffusion assay (Figure 2). The lower yield of Lac^+^ revertants on the plate for *recA* vs. wt QP5 may be due to increased killing of *recA* mutants by 5-azaC (evident by the ring of clearing in the disk diffusion assay and in Figure 3).

In *recB* mutants, the effect of 5-azaC was difficult to assess because the constitutive rate of QP5 Lac^+^ reversion in the absence of drug was already quite high, 13-fold higher than in wt strains (Figure 5). 5-Azacytidine treatment did induce mutagenesis further, by an additional factor of 3. Therefore, template-switch mutagenesis on the leading strand, including that induced by 5-azaC, does not depend on double-strand break repair. We cannot rule out, however, that some fraction of the mutagenesis does occur during DSB repair, in addition to a DSB repair-independent process. The elevation of constitutive mutation rates by *recB* but not *recA* may be due to increased levels of fork regression (addressed in the Discussion below).

With the lagging strand reporter, QP6, 5-azaC had a modest effect on mutation rates (9-fold stimulation) and its effect in *recA* mutants was even stronger, at 32-fold higher than untreated cells. Mutants in *recB* also showed a 5-fold higher constitutive rate of mutagenesis detected with QP6, less than the effect seen with the leading strand reporter, QP5. Induction of mutagenesis by 5-azaC with the QP6 leading strand was 2-fold.

### 3.3. The SOS Response Is Antimutagenic for Template-Switch Mutations

The DNA damage response in *E. coli*, known as the “SOS response”, is induced by the cleavage of a transcriptional repressor, LexA, promoted by the RecA-ssDNA filament [36]. We used a non-cleavable allele of LexA, *lexA3*, to block induction of the SOS response and measured rates template-switch mutagenesis using the QP5 *lacZ* reporter strain. The *lexA3* allele caused a high constitutive rate of mutagenesis, 9-fold higher than wt; and an even higher induction of mutagenesis by 5-azaC, 95-fold above its already high constitutive rate (Figure 5). Strains carrying *lexA3* also showed poorer survival after 5-azaC treatment (Figure 3), as reported previously [31]. As was seen with *recB* strains, mutagenesis detected with the lagging strand reporter QP6 in *lexA3* mutants, was modestly enhanced constitutively (4-fold) and slightly induced further by 5-azaC (3-fold). The SOS response therefore acts both to aid survival to 5-azaC and to prevent template-switch mutagenesis, especially on the leading strand.

### 3.4. Replication Mutants and Template-Switching

**Figure 6:**
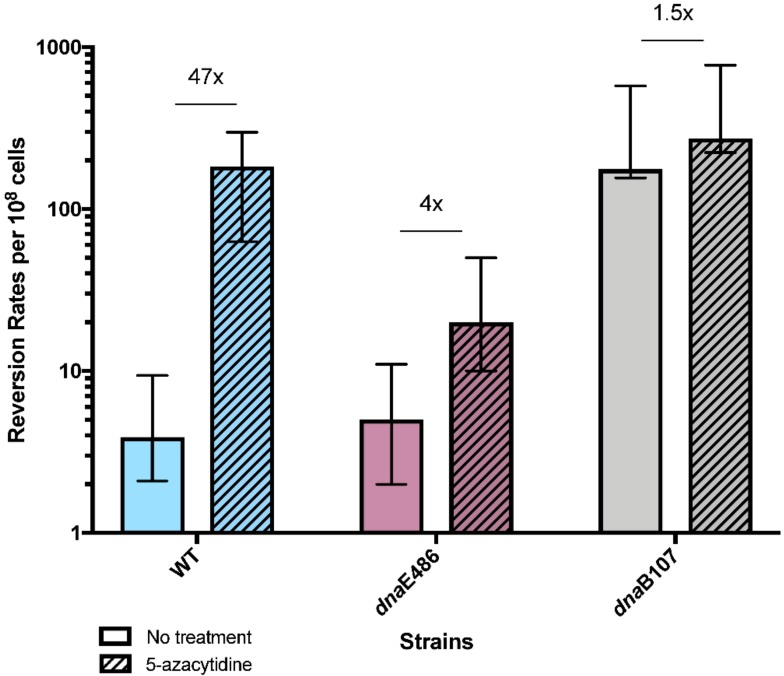
Reversion rates of *lacZ* quasi-palindromic (QP) WT, *dnaE486* and *dnaB107* leading strand mutational reporter after treatment with 5-azacytidine. Data show reversion rates per 10^8^ cells from multiple trials performed in different days. Error bars represent 95% confidence interval. Reversion rates of *lac*Z QP wild-type, *dnaE486*, and *dnaB107* mutant strains, grown at 30 °C, with and without treatment with 25 µg/mL of 5-azacytidine. Rates for no treatment are 3.9, 5.0, and 176, respectively. Rates with 5-azacytidine are 183, 20, and 273, respectively. Fold change comparing rates of reversion with and without exposure of 5-azacytidine is indicated above bars, and are 47, 4, and 1.5, respectively.

We tested mutations in two essential components of the replication machinery: *dnaE*, encoding the polymerase subunit of the replicative DNA polymerase, DNA Pol III, and *dnaB*, encoding a hexameric helicase that unwinds DNA at the replication fork. The *dnaE486* allele (S885P) causes a temperature-sensitive replication defect. At its permissive temperature, it gives rise to a mutator phenotype for general base substitution mutations. This mutator phenotype is relieved by loss of *dinB* (error-prone translesion synthesis DNA polymerase IV) [37], suggesting mutant Pol III competes poorly with Pol IV for access to the replication fork. We find that for template-switch mutagenesis induced by 5-azaC, *dnaE486* causes an *antimutator* effect, reducing mutagenesis 11-fold relative to wild-type cells (Figure 6). This result suggests that DnaE486 Pol III lacks some function required for template-switching induced by 5-azaC. The *dnaE486* allele had little effect on constitutive template-switch mutagenesis.

The *dnaB107* allele likewise causes temperature-sensitive DNA replication. At its permissive temperature, *dnaB107* mutants show elevated rates of genetic recombination [38], genomic rearrangements [18,19] and increased fork regression [39], suggesting that the mutant helicase is prone to dissociation from the fork. In the *dnaB107* mutant, at its permissive temperature of 30 °C, constitutive rates of reversion measured with QP5 were greatly elevated, 41-fold higher than wt strains (Figure 6). Rates after 5-azaC treatment were only slightly higher, at 1.5 above baseline rates.

### 3.5. Template-Switching Is Induced by Other DPC-Inducing Agents

**Figure 7:**
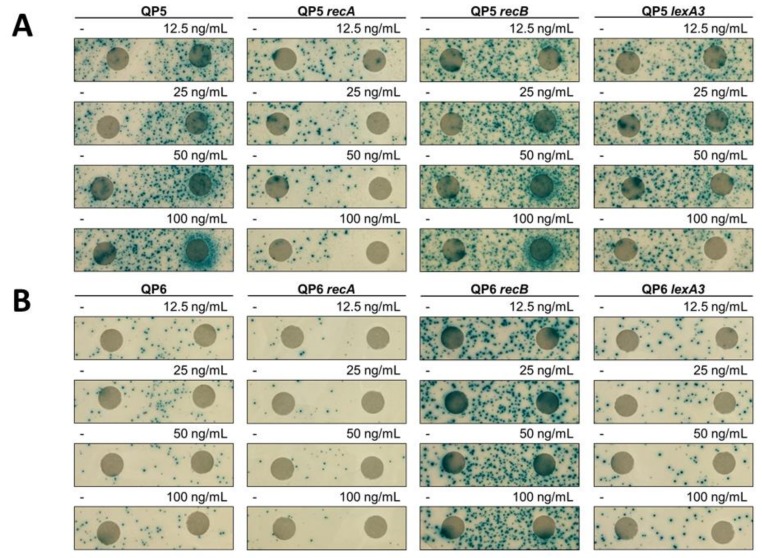
Mutator effect of ciprofloxacin. Disk diffusion assay for Lac reversion using QP5 (**A**) and QP6 (**B**) reporters ± *recA*, *recB*, *lexA3* and lactose X-gal papillation medium. Each disk on the right was saturated with 5 μL of ciprofloxacin at the indicated concentrations. The control disk on the left was spotted with 5 μL of water.

**Figure 8:**
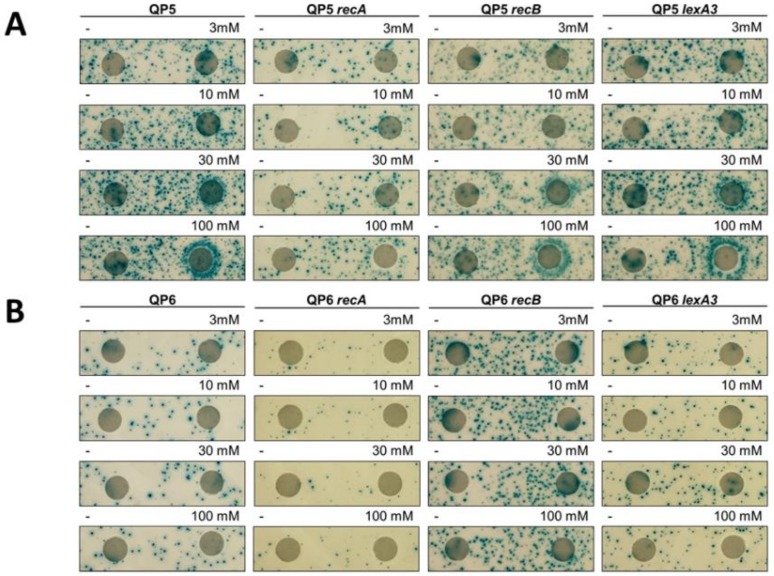
Mutator effect of formaldehyde. Disk diffusion assay for Lac reversion using QP5 (**A**) and QP6 (**B**) reporters ± *recA*, *recB*, *lexA3* and lactose X-gal papillation medium. Each disk on the right was saturated with 5 μL of Formaldehyde at the indicated concentrations. The control disk on the left was spotted with 5 μL of water.

**Figure 9:**
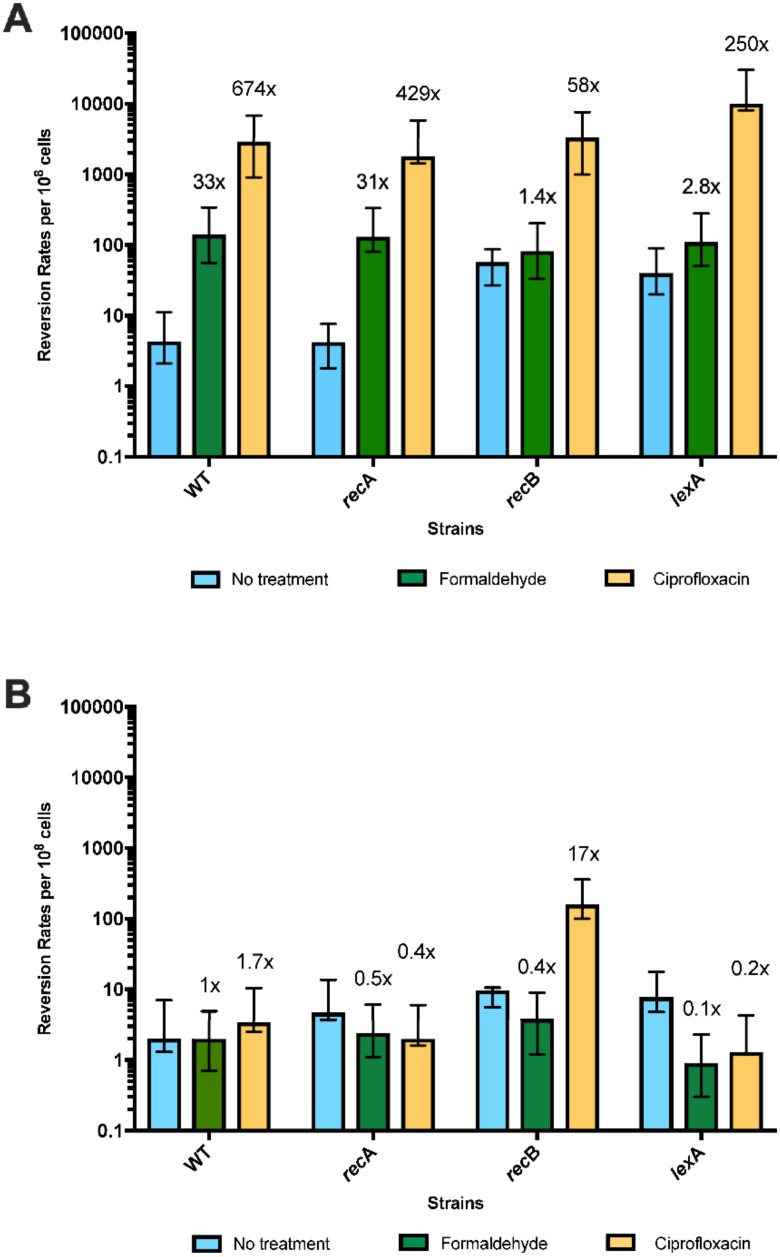
Comparison of reversion rates of *lacZ* QP leading (QP5) and lagging strand (QP6) mutational reporters after treatment with formaldehyde and ciprofloxacin. Data show reversion rates per 10^8^ cells from at least 6 multiple trials performed in different days. Error bars represent 95% confidence intervals. Reversion rate of *lac*Z wild-type and mutant reporters after exposure of 25 ng/mL of ciprofloxacin and 1 mM of formaldehyde. *(**A**)* Rates for QP5 reporters are 4.3, 140, 2900, 4.2, 130, 1800, 57, 82, 3300, 40, 110, and 10,000. Fold change comparing rates of reversion with and without exposure are 33, 674, 31, 429, 1.4, 58, 2.8, and 250 respectively. (**B**) Rates for QP6 reporters are 2, 2, 3.4, 4.7, 2.4, 2, 9.6, 3.8, 160, 7.8, 0.9, and 1.3. *Fold change* comparing rates of reversion before and after exposure are 1, 1.7, 0.5, 0.4, 0.4, 17, 0.1, and 0.2, respectively.

We tested two other treatments that induce DPCs for effects on template-switch mutagenesis. Formaldehyde nonspecifically crosslinks proteins to DNA, whereas ciprofloxacin is a topoisomerase II poison that traps the cleaved complex intermediate. Both formaldehyde and ciprofloxacin induced template-switch mutagenesis on the leading strand, detected with strain QP5, in the disk diffusion assay (Figure 7 and Figure 8). As with 5-azaC, effects of formaldehyde and ciprofloxacin on mutagenesis on the lagging strand, with the QP6 reporter, were not evident. Quantitative assays measuring rates of mutagenesis in split cultures after a single 2-h dose of formaldehyde or ciprofloxacin showed large increases in mutagenesis, 33-fold and 674-fold, respectively, (Figure 9) with the leading strand reporter QP5. As with 5-azaC, the mutagenic effects of formaldehyde and ciprofloxacin were independent of *recA* and *recB*. With the lagging strand reporter, QP6, formaldehyde and ciprofloxacin had less than a 2-fold effect, although some induction of mutagenesis was evident in the *recB* strain with ciprofloxacin.

## 4. Discussion

Mutations arising in imperfect inverted repeats, “quasipalindromes (QPs)”, have been noted in a wide range of organisms, from bacteria to humans (reviewed in [16,17]). As has been shown for both yeast and bacteria, these sites constitute hotspots for mutation [40,41]. Mutations at these sites universally perfect the palindromic sequence, suggesting a templated mechanism, whereby one arm of the repeat templates synthesis of the other [42]. A template-switch mechanism was confirmed by our genetic analysis of one mutational hotspot in the *thyA* gene of *E. coli* [43].

Many mutations arise during by improper base selection by DNA polymerases or by DNA lesions that promote miscoding. Templated-mutations, such as those at QP sites, are fundamentally different in nature because they are produced, not by coding errors by the polymerase, but rather by errors in alignment of the nascent strand with its DNA template. Transient cessation of replication with dissociation of the polymerase from the nascent strand, in theory, provides increased opportunity for this misalignment to occur. Furthermore, repair reactions at a blocked fork may promote misalignment and template-switching through the unwinding of the nascent strand. Because they block or stall DNA replication, compounds that are not general mutagens for miscoding-generated mutations may indeed be mutagens for template-switch generated mutations. Azidothymidine, a chain-terminating nucleoside, is one such compound: It strongly induces template-switch generated mutations with no detectable effect on general base substitutions or frameshift mutations [20]. Compounds that promote DPCs, such as 5-azacytidine, formaldehyde and topoisomerase II poisons, such as ciprofloxacin, are likely also in this category. With genetic reporter strains that specifically detect template-switch mutations, we show here that these compounds are highly mutagenic at sublethal doses. A number of other genetic rearrangements and mutations are promoted by template-switching and it remains to be determined whether they are likewise induced by DPCs. 

The dependence of 5-azaC induced mutagenesis on *E. coli*’s DNA cytosine methylase implies that it is the covalent Dcm:DNA complex trapped by 5-azaC that promotes template-switching. (The function of Dcm, which methylates the 2nd cytosine in CC[A/T]GG sequences in *E. coli*, is not entirely clear, but it may provide protection from plasmid-encoded restriction enzymes [44]). 5-Azacytidine also induces G:C to C:G transversions [45], an effect in *E. coli* dependent on Dcm [Lovett, unpublished results] and at CpG methylation sites in mammals [46]. It has been proposed that the chemical instability of 5-azaC:methylase adducts leads to ring-opening of cytosine and subsequent mispairing of the ring-opened lesion with cytosine [46].

The mode of action of the other agents in this study is less certain, although the similar of genetic effects on their mutagenicity to that of 5-azaC, revealed here, suggests a role for DPCs in mutagenesis. Formaldehyde produces a wide variety of lesions that may contribute to mutagenesis. Ciprofloxacin specifically inhibits type II topoisomerases [47] for which *E. coli* has two, DNA gyrase and Topoisomerase IV, trapping the covalent cleaved complex [48]. Ciprofloxacin inhibits DNA replication and we do not know whether its mutagenic effect is due to general replication inhibition rather the production of DPCs. Further work should clarify these mutagenic effects. Although ciprofloxacin is a specific inhibitor of the bacterial type II topoisomerases, mammalian topoisomerase II inhibitors with similar modes of action, such as etoposide and doxorubicin, are commonly used in cancer chemotherapy [49].

**Figure 10:**
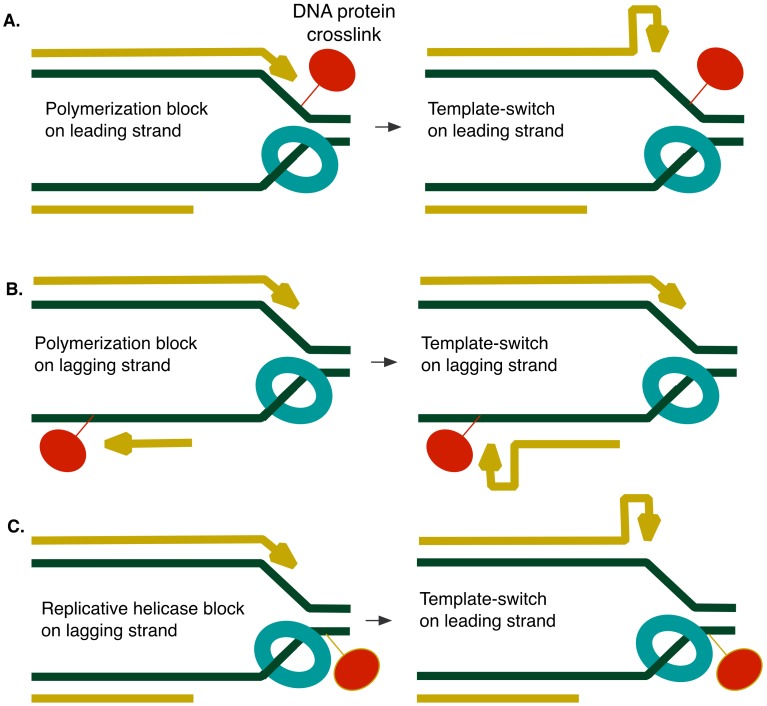
DPC blocks at the replication fork and consequential template-switching. (**A**) Polymerization block (red) on the leading strand promotes template-switching on the leading strand. (**B**) Polymerization block (red) on the lagging strand promotes template-switching on the lagging strand. (**C**) Fork helicase block (red) on the lagging strand promotes template-switching on the leading strand.

We find that 5-azaC stimulates template-switching more strongly on the leading strand than on the lagging strand. We imagine that trapped complexes on either strand can impede DNA polymerization (Figure 10A,B), providing time for the nascent strand to misalign with itself. The preferential effect on the leading strand, however, may result from blocks to progression of the fork helicase, DnaB, which, in bacteria, encircles the lagging-strand template [50]. In such an instance, sealing of the lagging strand progresses, but a 3’ end persists on the leading strand, giving it additional opportunity to isomerize to the mispaired configuration (Figure 10C). Mutations to the DnaB helicase that reduce its efficiency or stability, such as *dnaB107*, likewise would promote template-switching on the leading strand, explaining the large increase in constitutive mutation rates seen in *dnaB107* strains. Leading strand template-switching may also be promoted by fork regression (see Discussion below and Figure 9), known to be elevated by *dnaB* mutations [39].

We establish here that an allele affecting the polymerase activity of DNA Polymerase III diminishes the level of template-switch mutagenesis induced by 5-azaC. In prior unpublished work, we had also seen that *dnaE486* reduced template-switching at the *thyA* mutational hotspot. This finding implicates some function of DNA Pol III in template-switching; alternatively, template-switching in *dnaE486* strains may lead to lethality so that reversion mutants cannot be recovered.

Induction of the SOS DNA damage response is not only protective for lethality associated with DPCs but also for template-switch mutagenesis induced by DPCs. We observed large increases in constitutive and 5-azaC-induced mutagenesis in *lexA3* strains, in which the SOS response cannot be induced. The specific SOS functions [36] responsible for this effect are unknown but might include one or more of three translesion synthesis (TLS) DNA polymerases, Pol II, Pol IV or Pol V. Our prior study of the *thyA* template-switch mutational hotspot provided evidence that the TLS polymerases act in some redundant fashion to reduce the likelihood of template-switch mutagenesis [43]. Additional SOS-regulated genes may also play a role.

**Figure 11:**
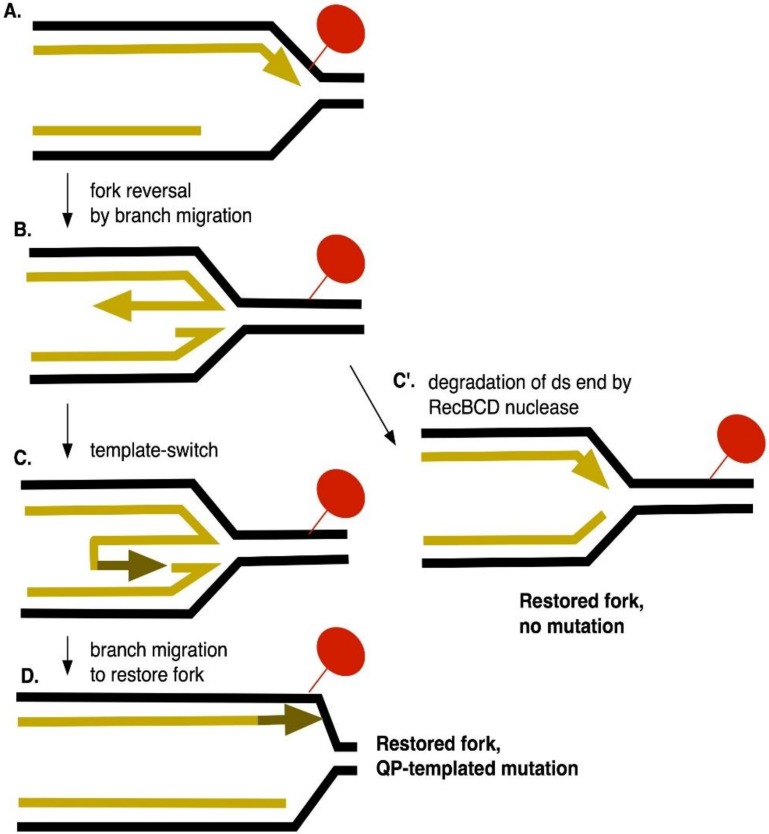
Fork regression, leading strand template-switching and the role of RecBCD nuclease in mutation avoidance. (**A**) Polymerization is stalled by a leading strand DPC. (**B**) Branch migration, potentially mediated by DNA helicases or RecA, promotes regression of the fork and produces a 4-way junction, with a short double-strand end. (**C**) If the double-strand end persists, fold-back of the nascent 3’ leading strand end, primes synthesis, causing a template-switch. D. After branch migration to restore a fork structure, the newly templated DNA is retained, giving rise to a QP mutation. C’. Alternatively, RecBCD exonuclease degrades the extruded strand, resetting the fork backwards and removing the potential for mutation.

DPCs can lead to the formation of double-strand breaks in mammalian cells [51]. In bacteria, tolerance of DPCs is diminished in *recA* and *recBCD* mutants [31,32,33,34,35], strains defective in DSB repair. RecA is not, however, required for the recovery of template-switch mutations induced by 5-azaC, formaldehyde or ciprofloxacin. Interestingly, *recB* mutants exhibit very high constitutive rates of template-switch mutagenesis, a phenotype not shared by *recA* mutants. Therefore, RecBCD provides some function, other than its role in DSB repair, that limits template-switching during normal growth. We think a likely explanation is RecBCD’s ability to degrade and reset a regressed replication fork, a property proposed by Michel and collaborators [39] (see Figure 11). Branch migration of a blocked replication fork produces a 4-strand Holliday junction, with a short linear end. RecBCD, with its potent exonuclease activity, should attack this end, thereby resetting the fork to a 3-strand fork structure. If the double-strand end at the regressed fork persists, as in *recB* mutants, the 3’ strand at the end may have the opportunity to fold back upon itself to initiate DNA synthesis, resulting in a template-switch mutation after the fork is reestablished (Figure 11). The structure of the regressed fork constrains template-switching to the leading strand, explaining the stronger effects on mutagenesis detected with leading strand reporter strain than the lagging strand reporter strain. Fork regression has not been previously considered to be mutagenic, except in its propensity to produce DSBs, and fork regression may prove to be a significant source of template-switch mutations.

DPC-promoting agents such as 5-azac 2′-deoxy cytidine (Decitibine) and Topoisomerase II poisons are used for cancer chemotherapy and this study reveals a potential genotoxic consequence to their use in humans. 

## Figures and Tables

**Table 1:** *Escherichia coli* K-12 strains used in this study.

Strain	Genotype	Origin or Reference
MG1655	F^-^ *rph-1*	
STL7180	*recA::cat*	Lab collection
STL12071	*lexA3 malF::*Tn*10 kan*	Lab collection
STL15144	*lacZ(QP5) lexA3 malF::*Tn*10kan mphC281::*Tn*10*	This work
STL15654	*lacZ(QP6) mphC281::*Tn*10*	Lab collection
STL16519	*lacZ(QP6) lexA3 malF::*Tn10 *kan mphC281::*Tn*10*	This work
STL17685	*lacZ(QP5) mphC281::*Tn*10*	Lab collection
STL18747	*lacZ(*QP5*) dcm∆::*FRT *kan mphC281::*Tn*10*	This work
STL18749	*lacZ(*QP6*) dcm∆::*FRT *kan mphC281::*Tn*10*	This work
STL19829	*lacZ(*QP5*) recA::cat mphC281::*Tn*10*	This work
STL19831	*lacZ(*QP6*) recA::cat mphC281::*Tn*10*	This work
STL19843	*recB∆::*FRT *kan*	This work
STL20891	*lac*Z(*QP5*) *dnaE486 zae-3095*::Tn*10kan mphC-281::*Tn*10*	This work
STL21742	*dcm∆::*FRT *kan*	This work
STL21922	*lac*Z(QP5) *dnaB107*(ts) *malE*::Tn*10kan*	This work
STL21926	*lacZ(*QP5*) recB∆::*FRT *kan mphC-281::*Tn*10*	This work
STL22017	*lacZ(*QP6*) recB∆::*FRT *kan mphC-281::*Tn*10*	This work

All strains are MG1655 derivatives, constructed by P1 transduction. ∆FRT *kan* alleles were derived from the Keio knock-out collection [28]. Constructions were confirmed by PCR analysis or testing for known phenotypes.
